# Development of an online clinical trial recruitment portal for the NIHR mental health BRC

**DOI:** 10.1186/s40900-016-0024-0

**Published:** 2016-04-01

**Authors:** Sarah Markham

**Affiliations:** KCTU, Department of Biostatistics, IoPPN, King’s College London, London, UK

**Keywords:** Clinical, Trials, Participant, Recruitment, Online, Portal

## Abstract

**Plain English summary:**

In order to test whether or not new treatments for mental health disorders help patients get better according to clinician/patient selected criteria, it is often necessary to test them on patients under safe, carefully monitored conditions called clinical trials. It is necessary to find enough patients to take part in a clinical trial so that the results of the trial are reliable. The NIHR Mental Health BRC (here after abbreviated to BRC) is a centre for research in London which seeks to find out better ways to treat patients with mental health difficulties. The BRC has experienced problems trying to find sufficient numbers of patients to participate in its clinical trials as it appears that insufficient patients were being told by their doctor about opportunities to participate in clinical research. In order to help the BRC find enough patients to volunteer to take part in its clinical trials, the author (a patient representative) of this article and a clinical researcher in the nearby Institute of Psychiatry, Psychology and Neuroscience (IoPPN) decided to work together to try to find the best way to let patients know more about what clinical trials are, what it is like to take part in them and which clinical trials are seeking patients to take part.

The author and researcher used a report by the Association of Medical Research Charities (AMRC) on patient difficulties in finding clinical trials to take part in, and the recommendations it made, to guide them in building a website to give such patients the information on clinical trials they wanted (including clinical trials run by the BRC).

The author and researcher also asked patients, carers, staff at the IoPPN and BRC what they thought of the website and how to make it better. They used the ideas, suggestions and criticisms to improve the website. The author and researcher also asked mental health charities and research organisations if they would advertise the final version of this website on their own websites; many said yes, they would. The manager of the BRC on reviewing the website, agreed that a final version of the website with the NIHR Mental Health BRC logo would be paid for and will form part of a new main website for the BRC in early 2016.

**Abstract:**

ᅟ

**ᅟ:**

Public & patient recruitment to clinical trials is viewed as one of the main barriers to the implementation of clinical trials. This difficulty is often attributed to the working culture of the NHS, rapid turnover of staff and patients and poor-gatekeeping in referring patients to suitable clinical trials. In response to the recruitment difficulties experienced by the Psychosis Studies Clinical Academic Group at the NIHR Mental Health Biomedical Research Centre, Denmark Hill, London, a member of the Office of Psychosis Studies at King’s College London and a member (the author) of the King’s Clinical Trials Unit, King’s College London developed an initiative to create an online clinical trial recruitment portal/information hub for the NIHR Mental Health BRC. The primary purpose of this initiative being to promote patient and public awareness of and interest in participating in clinical trials.

## Correspondence/Findings

### Clinical trials: background information

The World Health Organization (WHO) defines a clinical trial as any research study that prospectively assigns human participants or groups of humans to one or more health-related interventions to evaluate the effects on health outcomes [1]. Interventions may include, but are not restricted to drugs, cells and other biological products, surgical procedures, radiologic procedures, devices, behavioural treatments, process-of-care changes, preventive care, etc. [2].

Clinical trials test the safety and efficacy of medical treatments, providing the body of scientific evidence on which the pharmaceutical industry is built. Human medicines cannot be sold without permission from a licensing authority and permission will not usually be granted unless a clinical trial has demonstrated the medicine’s success in treating the condition for which it will be marketed [3]. Clinical trials, in addition to generating valuable scientific evidence, also provide patients with an important way of accessing products that have not yet reached the market, offering hope to those for whom existing treatments have failed. The necessity for a clinical trial to be performed before a medicine can gain regulatory approval means that trials are also big business [4].

Medical research in mental disorder is needed to answer questions that are important to people affected by mental health problems, their families and friends, and the people working to support them across the health and social care sector and beyond [5]. Mental ill-health impacts on all aspects of peoples’ lives and medical research can make a tremendous positive impact on those who suffer from mental disorders through the development of new and better medicines and therapeutic techniques and technology [5]. This in turn can benefit the whole of our society by generating social and economic benefits that contribute to thriving communities. Research provides hope that those in future generations experiencing mental health problems will have a better quality of life and place in society [5].

According to the Mental Health Foundation:

- Mental health problems account for a quarter of all ill health in the UK yet they get less than 6 % of all health research funding.
- 1 in 10 young people are affected by mental health issues.
- Currently 75 % of those with a mental health problem receive no treatment or support.
- Suicide is the most common cause of death in young men.

The social and economic cost of mental health problems in England alone is £105 billion and UK businesses lose £26 billion a year due to mental health problems in the workforce [6].

Current psychiatric treatments are only partially adequate and certain psychopharmacological modalities carry unpleasant or even severe side-effects such as sedative adverse reactions and immunological dysfunction [7]. As in any branch of medicine, participation in clinical research studies or trials might provide a patient with the opportunity to access better (more effective and with milder adverse reactions) treatment.

There is a clear case for the need for mental health research. This research often needs clinical trials to be performed, to safely and rigorously identify and test better treatments for those suffering from mental disorders. Without mental ill-health sufferers who are willing to participate in clinical trials such as the ones performed at the BRC, no progress can be made.

### Participant recruitment to clinical trials

The NIHR Mental Health BRC (http://brc.slam.nhs.uk/) is one of eleven NIHR funded Biomedical Research Centres throughout England. It is hosted and run by the Institute of Psychiatry, Psychology and Neuroscience (IoPPN) King’s College London and the South London and Maudsley Foundation Trust (SlaM). Also known as the Maudsley BRC, it values those who are willing to give up their time to participate in the clinical trials they run and are eager to show their appreciation. Prospective participants are given the fullest information about what they can expect from a clinical trial and what it will require of them before they sign a consent form. In return they are kept fully informed of their progress during the clinical trial process and remunerated for their time and any travel expenses incurred. If they wish to withdraw from the trial at any time they may do so, as enshrined in law through acceptance of the Helsinki Declaration [8]. On withdrawal from participation in a clinical trial, a patient will be offered the standard care/treatment which applies normally to any patient in that situation.

In many cases participants have the option of receiving information about the outcomes of the trial when they are finalised. Everyone who participates helps towards developing a better understanding of mental disorder and how it may be more effectively treated.

Public and patient recruitment to clinical trials is one of the main barriers to the implementation of clinical trials. Many clinical trials fail to recruit the desired number of public and/or patient participants [9]. According to research conducted by the author for the King’s Clinical Trials Unit (KCTU), IoPPN, King’s College London and, there are several causal factors all of which are identified in the published paper ‘Promoting Factors and Barriers to Participation in Early Phase Clinical Trials’ [10], the main three being:

- cultural resistance in the NHS (Often perceived as a consequence of the relational dynamics between academics and clinicians)
- rapid turnover of clinical teams and patients
- as gatekeepers, clinicians may seek to protect people for whom they provide care from the perceived burden of research participation and/or an intervention perceived to be futile for an intractable condition.

Furthermore clinical trials need to recruit to capacity as, in general larger numbers of participants leads to more representative and informative statistical analysis of clinical trial data and thereby, potentially the identification and development of more effective psychiatric treatments [11]. It is often important for clinical trial participants to be of a diverse nature whilst still conforming the exclusion/inclusion trial specific criteria. Again this is optimised by maximising recruitment and retention to clinical trials.

The BRC had identified a paucity of referrals from clinicians as impacting negatively on recruitment to clinical trials. This was viewed as a longstanding problem and seemingly resistant to resolution. It was decided to improve the BRC website in order to attract more public and patient awareness of and interest in participating in clinical trials and thereby encourage direct expressions of interest in clinical trial participation. As all prospective trial participants are screened thoroughly before being given the opportunity to consent to participate, the absence of a clinical referral would not affect patient (or healthy volunteer) safety or well-being.

The UK Clinical Trials Gateway is the NIHR-hosted website that allows people to search for information about clinical trials in the UK (http://www.ukctg.nihr.ac.uk/default.aspx).

However according to a public survey [12] conducted by the AMRC - Association of Medical Research Charities (designed to ascertain the accessibility of the UK Clinical Trials Gateway and information on currently recruiting clinical trials:

- 80 % had not heard of UKCTG before receiving the survey.
- Only 28 % had taken part in a clinical trial.
- 38 % said they knew little or nothing about clinical trials and would like a clear and reliable source of information to learn more; 56 % said it would help them or someone they care for explore opportunities to take part in a clinical trial now or in the future.
- 64 % said they would like to find out about trials recruiting in their local area.

This indicates that there are significant numbers of people who want to know more about clinical trials and how to find one relevant to their needs.

The survey was carried out through *surveymonkey* and a link distributed via email to a range of patient groups and networks. It was also posted on a number of websites including the NIHRs Clinical Research Network, INVOLVE and UKCTG. The survey ran for the whole of July 2012.

As a result of these findings the AMRC made the following recommendations to help the UKCTG better reflect the priorities of people interested in finding out about, and taking part in, clinical trials:

**Vision and strategy** - Going forward the UKCTG needs a more patient-focused strategy so that it meets their needs as well as the UK research community’s.

**Awareness and promotion** - the site needs renaming and made easier to find by patients searching for information. It needs better promotion by the NHS, by charities and patient groups, research funders and other organisations.

**Public Involvement/User Panel** - a user panel should be involved in the future development of the site, including advising on the best way to implement the above recommendations and to build up a knowledge base of public experience of using the UKCTG and taking part in trials, to inform patients and the wider research community.

In order to resolve the BRC’s public and patient clinic trial recruitment difficulties the researcher (a member of the BRC’s Psychosis Clinical Academic Group) was appointed to spear-head an initiative to improve access to information and participation in currently recruiting clinical trials. The researcher recruited the author to the initiative after the latter expressed an interest in being involved and proffered some of her own knowledge and research findings on the issue.

At the time the author began working with the researcher, she was aware of the AMRC survey, its findings and recommendations. This persuaded the researcher and author that in order to enhance clinical trial recruitment at the BRC it would require a dedicated online clinical trial recruitment portal with the following features:

- **Vision and Strategy** – The clinical trial recruitment portal would feature current, clear, up to date, easy to understand information on the nature and purpose of clinical trials and experience of participating in a clinical trial.
- **Awareness and promotion** - In order for the portal to become known to interested patients and members of the public, it would be advertised on major mental health charity/research websites, e.g. MQ, Rethink, NSUN, etc.
- **Patient Use/Involvement Panel** - The entire function and structure of the online clinical trial recruitment portal would be discussed with the BRC Service User Advisory Group (SUAG) of which the author is a member, and their recommendations incorporated into the beta version of the online recruitment portal. This occurred on 05/08/15. This group remains a valuable consultation resource for the online clinical trial recruitment portal.

## Chronology of events

### March-April 2015

The researcher and author meet to discuss how to improve the clinical trial pages of the website. The author gives information on national and global research findings that service users find it hard to access information on recruiting clinical trials and the clinical trial process [12, 13].

### 21/04/15

The researcher and author meet with the manager of the BRC to discuss how to modify and improve the clinical trial pages of the BRC website.

### May-June 2015

The author in consultation with her colleague, the researcher, builds a beta version of an online clinical trials recruitment portal for the BRC as a means of conveying their shared vision of how to promote public and patient participation. The author uses *Joomla*, an open-source content management system to construct the portal [14].

### 08/06/15

The researcher and author meet with a senior researcher in the NIHR Mental Health BRU (Biomedical Research Unit) to discuss the active online recruitment portal for senior researcher’s PROTECT study of ageing in adults over 50 years old [15]. The PROTECT study portal has proved very successful in recruiting patients to participate. There is consideration of the various features and functionality of the PROTECT study portal together with the possibility of extending the PROTECT study portal to the BRC or re-using the code to create an independent online platform. The author after later consultation and discussion with the researcher, modifies the beta online clinical trial recruitment portal to include a template functionality modelled on the PROTECT study portal. This would allow research groups to develop their own study specific online recruitment portal, and link it to the main BRC online recruitment portal.

### 10/06/15

Presentation of the beta version of the clinical trials recruitment portal at the Clinical Disorders Cluster Group Board Meeting with subsequent discussion/feedback session. The consensus view at the meeting is that the beta version is potentially very useful in encouraging participation in clinical trials and that its formal development should be explored together with service user and carer input. Subsequently the author seeks competitive bids sought for the contract for the final version.

### 01/07/15

The researcher and author meet with prospective developers for the final version of the clinical trials recruitment portal. They discuss possible design specification and the associated cost of developing and maintaining a final version of the recruitment portal. Quotes for the work are requested.

### July-August 2015

Feedback on beta website requested from the RCPsych and national mental health charities (*Mind, Sane, Rethink*, *MQ, McPin, NSUN)* and their associated service user groups/membership. The organisations send out (by email and post) details of the intended purpose of the beta website to their members and service user groups, together with a hyperlink to the website and requests for feedback to be emailed or posted directly to the author. The author receives the feedback which is considered and used to amend and extend the beta website. Overall the feedback is complimentary, but there are requests for a clarification of acronyms featured on the website and a clearer classification of the kinds or clinical trials run by the BRC. The author acts to clarify and minimise the use of acronyms and regroups the list of currently recruiting clinical trials at the BRC according to the specific mental disorders concerned. A member of the RCPysch’s Carers’ Forum reports by email that the beta website is easy to navigate and requests that a summarised guideline to the process of participation in clinical trials is provided. This is actioned at once by SM. *MQ*, *McPin* and *NSUN* report they will be agreeable to consider hosting a link to the final version of the online recruitment portal on their respective websites on reviewing the finished branded NIHR Mental Health BRC online recruitment portal for themselves. *Mind*, *Sane* and *Rethink*, reply that they only advertise their own branded products on their website but they are agreeable to the NIHR Mental Health BRC posting updates and references to the final online recruitment portal on their respective *Facebook* pages.

### July-August 2015

Feedback on beta website requested from all BRC principal investigators. The feedback, is uniformly positive from those who respond. This feedback, together with that obtained via the various mental health charities, is collated to present and discuss at the Experimental Medicine & Clinical Trials Theme Meeting on 07/09/15.

### 05/08/15

Consultation with the NIHR Mental Health BRC Service User Advisory Group to gain more patient and carer views on the online clinical trial recruitment portal; the information and services it should provide and how these might best be presented using the portal. Strong interest is expressed in the online recruitment portal and the overall feedback is positive. No significant changes are requested, but the importance of all information on upcoming and currently recruiting clinical trials being up to date is emphasized.

### 07/09/15

Presentation of the beta version of the online clinical trial recruitment portal at the Experimental Medicine and Clinical Trials Theme Meeting together with associated feedback as detailed above. Approval is given for a final version of the portal to be created using the services of a professional software engineer and incorporated into the main NIHR Mental Health BRC website, providing funding can be found.

### 22/09/15

Presentation of the beta version of the online clinical trial recruitment portal at the Clinical Disorders Cluster Group Board Meeting together with associated feedback. There is subsequent discussion/feedback session. Approval for a final version of the portal to be created using the services of a professional software engineer and incorporated into the main NIHR Mental Health BRC website is confirmed, providing funding can be found.

### 01/10/15

Meeting of the author and researcher with the manager of the BRC. The beta version of the online clinical trial recruitment portal with all modifications completed is presented and funding for the final NIHR Mental Health BRC branded version discussed. It is agreed that the contract for the final version will be awarded to *Limetree Products Ltd* [16]. It is also agreed that the final version will be paid for by the core BRC funding as part of a wider project to update and modify the entire BRC website (to enhance its accessibility and relevance). Thus funding for the final (branded) version of the online clinical trial recruitment portal is secured.

## Development details

The initiative was limited somewhat by the length of time it took for certain NIHR Mental Health BRC stakeholder groups to respond to the requests for feedback on the beta version of the online clinical trial recruitment portal. This is believed to be due to annual leave and heavy work schedules of those stakeholders.

The potential cost for a professional software engineer to create the final online clinical trial recruitment portal as a stand alone online facility would be ~ £14,000 plus possible maintenance costs of ~ £1000 per annum. This possible cost has been reduced by negotiation between the manager of the BRC and *Limetree Products Ltd* with regard to including the creation of the final version of the online clinical trial recruitment portal in the *Limetree Products Ltd’s* contract for the creation of the new BRC website (based on a modification of the current BRC website).

The social implications of the initiative are potentially enhanced public and patient awareness of the clinical trial process and nature of participation in clinical trials, together with enhanced public and patient access to up to date information on upcoming and currently recruiting clinical trials. This is in line with the recommendations made to the UK Clinical Trials Gateway to promote public and patient awareness and access to participation in clinical trials [12].

## Recruitment methods of other NIHR BRCs

The BRC is the first of the 11 NIHR BRCs to develop a clinical trials recruitment portal/information hub. The other 10 NIHR clinical trials use traditional methods to recruit participants, e.g. referral by front-line clinicians, printed poster and newspaper advertisements and listing the clinical trials in national and international registries and databases such as the WHO International Clinical Trials Registry Platform (http://www.who.int/ictrp/en/). However there are two exceptions of note:

1. NIHR Cambridge Biomedical Research Centre (http://www.cambridge-brc.org.uk/public) – which has an online pre-screening process for certain non-medication (minimal risk) trials run by the Cognition & Brain Sciences Unit.
2. NIHR Biomedical Research Centre at Oxford University Hospitals NHS Trust and the University of Oxford (http://oxfordbrc.nihr.ac.uk/) – which has the ‘Patients Active in Research’ website; an online submission form that allows service users to register ‘disorders of interest’ with regard to the nature of clinical trials they would wish to be notified of. There is also the option of specifying whether or not they would wish to be contacted directly by the corresponding research teams.

## Content and features of the beta version on the BRC online clinical trial recruitment portal

The beta version of the BRC Online Clinical Trial Recruitment Portal has the following features (which will all feature in the final branded version of the online recruitment portal). All features were either approved or requested by the various stakeholders we consulted including patients and carers:

- full (and summarised) account of the clinical trial process and the stages of participation including a glossary of terms relating to clinical trials
- motivation behind and experience of being a public/patient participant in a clinical trial plus how the BRC demonstrates its appreciation to volunteers for their participation
- provision of complete up to date details of all trials which the BRC is currently running and relevant contact details for those interested in participating
- links to national and international clinical trial search sites (and sources of further information on the clinical trial process, the BRC, SLaM, IoPPN and King’s College London together with an FAQs section
- information on the outcomes and impact of completed clinical trials
- online form to request information of upcoming currently recruiting trials relating to various clinical disorders as specified by the online website users
- online form to submit feedback on participant experience and opinions of the portal i self, the NIHR Mental Health BRC online clinical trial recruitment portal, etc.
- online form to submit questions on clinical trials
- current and upcoming public events at the IoPPN and NIHR Mental Health BRC
- news articles on clinical trials featuring stories from the IoPPN, SLaM and the wider world or clinical trials
- an RSS feed
- associated social media platforms (*Twitter* and *Facebook* webpages) together with an online community ‘Mental Health Clinical Trials’ hosted by *HealthUnlocked.com* [17].
- templates to be used by BRC research groups who wish to develop their own study specific online recruitment portal (linked to the main BRC portal)

In choosing and designing the above features the author drew on her own service user experience of seeking information relevant to the disorder she suffers from, on how to participate in clinical trials and also on what she would like an online clinical trial recruitment portal to offer her as a prospective participant. The researcher and the author also drew on the feedback gathered from mental health charities, various patient and carer groups, the SUAG and other BRC stakeholders together with research by the AMRC and CISCRP. Throughout the project the researcher’s and the author’s aim was to produce an accessible online clinical trial recruitment portal which the public and patients would find welcoming, attractive, informative, relevant to their interests and needs and which could be advertised on major mental health charity/research websites.

## Future development plans

Funding for the development and maintenance of the final version of the BRC online clinical trial recruitment portal has been secured and will come from the core BRC funding. This development has begun and is being performed by *Limetree Products Ltd*. A dedicated member of the BRC staff will be trained to routinely maintain and update the recruitment portal. Additional maintenance will be carried out by *Limetree Products Ltd* according to need. The branded ‘NIHR Mental Health BRC’ online recruitment portal will be launched in early 2016 and as a part of the main public BRC website will be free to access and not require a login or pre-registration.

## Postscript

The author is a mental health service user who entered into service user involvement work as part of her recovery from a significant suicide attempt. Prior to this her life had been one of academic achievement compromised by the difficulties she encountered as a consequence of an untreated mental disorder. Determined to rebuild the quality of her life, she entered into service user involvement as a means of making positive use of her experience of having a mental disorder and of being treated by mental health services. On being discharged from hospital she applied to become a member of the BRC’s Service User Advisory Group (SUAG). The SUAG is a group of mental health services users and carers, each of whom have academic experience and are able to provide research groups with informed service user and carer perspectives on research proposals presented to them at regular meetings.

Membership of the SUAG led to the opportunity for the author to become the patient representative of the Experimental and Clinical Trials Cluster Group; one of the four cluster groups comprising the BRC. Once accepted as the patient representative, she was instructed to develop the role as she thought best which led to her researching and compiling a report for the BRC on the barriers to the implementation of clinical trials in routine with clinical care and possible means of resolution; this being of relevance to the Experimental Medicine and Clinical Trials Cluster Group at the time [10].

The difficulties for interested potential participants in trying to find out how to take part in a clinical trial and which trials were currently recruiting, as reported by the AMRC [12], reflected the author’s experience as a mental health service user wanting to become a participant in medical research studies. Being the patient representative for the BRC’s Experimental Medicine and Clinical Trials Cluster Group led to the opportunity for the author to work with the BRC’s Clinical Disorders Cluster Group to promote public and patient awareness of clinical trials and enhance participation in clinical trials run by the BRC.
